# Changes in Plasma Sphingolipid Metabolites Following Roux‐En‐Y Gastric Bypass in Women With Obesity and Type 2 Diabetes: A Pilot Metabolomic Cohort Study

**DOI:** 10.1002/lipd.70019

**Published:** 2025-11-12

**Authors:** Gabriela de Oliveira Lemos, Raquel Susana Torrinhas, Natasha Mendonça Machado, Dan Linetzky Waitzberg

**Affiliations:** ^1^ Laboratory of Nutrition and Metabolic Surgery of the Digestive System – LIM 35, Department of Gastroenterology, Hospital das Clinicas HCFMUSP, Faculdade de Medicina Universidade de São Paulo São Paulo Brazil

**Keywords:** bariatric surgery, cholesterol, lipidomics, Roux‐en‐Y gastric bypass, sphingolipids

## Abstract

Although sphingolipids are key players in lipotoxicity and metabolic diseases, their response to bariatric surgery and their relation to metabolic improvement remain unclear. This pilot study investigated plasma sphingolipid remodeling after Roux‐en‐Y gastric bypass (RYGB) and its associations with clinical and biochemical markers of postoperative metabolic improvement in women with obesity and type 2 diabetes (T2DM). Plasma samples, anthropometric, body composition, and biochemical data (glucose, HbA1c, insulin, C‐peptide, and lipid profile) were collected from 30 participants before and 3 months after surgery. T2DM remission was defined according to ADA 2021 criteria. Plasma sphingolipids were identified using untargeted metabolomics, which involved ultra‐performance liquid chromatography coupled with mass spectrometry. Univariate and multivariate analyses were performed using Jamovi (version 2.2.5) and MetaboAnalyst (versions 5.0 and 6.0). RYGB led to reductions in body weight and anthropometric measures, with improved body composition. Patients demonstrated glycemic improvement, with 18 achieving remission of T2DM. The lipid profile also improved, with a decline in total cholesterol driven by reductions in pro‐atherogenic fractions. Among 32 plasma sphingolipids identified, 21 changed significantly after surgery. Sphingolipids showed strong‐to‐robust correlations with the lipid profile, particularly SM(d18:1/20:0) and SM(d18:1/22:0) with total cholesterol and LDL‐c after surgery, but moderate and poor correlations with body composition, and glycemic markers, respectively. Plasma sphingolipids underwent significant remodeling after RYGB, with strong associations with plasma cholesterol, particularly with SM(d18:1/20:0) and SM(d18:1/22:0). These findings suggest that specific sphingolipid species may contribute to or reflect plasma lipid adaptations to surgery and warrant further investigation as potential metabolic biomarkers.

**Trial Registration:** This protocol is part of a broader umbrella study registered at www.clinicaltrials.gov under the identifier NCT01251016

**Abbreviations**
ADA: American Diabetes Association
BMI: body mass index
Cer: ceramide
CV: coefficient of variation
FDR: false discovery rate
GEE: generalized estimating equations
HbA1c: glycated hemoglobin
HDL‐c: high‐density lipoprotein
LDL‐c: low‐density lipoprotein
PLS‐DA: partial least squares discriminant analysis
QC: quality control
RYGB: Roux‐en‐Y gastric bypass
SM: sphingomyelin
SPT: serine‐palmitoyl transferase
T2DM: type 2 diabetes mellitus
TLR4: toll‐like receptor 4
VIP: variable Importance in Projection
VLDL‐c: very‐low‐density lipoprotein

## 1 Introduction

Obesity and type 2 diabetes mellitus (T2DM) are major public health concerns, often coexisting and sharing pathophysiological mechanisms linked to lipotoxicity and chronic inflammation (Unger 2002). Metabolic and bariatric surgery, particularly Roux‐en‐Y gastric bypass (RYGB), has been recognized as a highly cost‐effective long‐term treatment for individuals with severe obesity and obesity‐related T2DM (Cummings and Rubino 2018). In addition to sustained weight loss, early glycemic control, and long‐term T2DM remission, RYGB also reduces the risk of cardiovascular events and cancer (Zhang et al. 2020; van Veldhuisen et al. 2022).

After RYGB, a decreased energy and fat intake follows weight loss (Miller et al. 2014), along with decreased plasma fatty‐acid availability and intestinal Toll‐like receptor 4 (TLR4) activation (Sala et al. 2020; Machado, 2018). Thus, the postoperative decrease in sphingolipids' abundance is anticipated, yet findings from RYGB studies suggest that these alterations differ according to the degree of lipid saturation (Vaz et al. 2022). Although under obesogenic conditions, sphingolipids have been implicated in metabolic dysfunctions, few studies have empirically investigated changes in circulating sphingolipid metabolites following bariatric surgery, particularly in relation to metabolic outcomes (Vaz et al. 2022). This gap is relevant for understanding the integrated biochemical adaptations induced by RYGB.

T2DM associated with obesity is characterized by chronically elevated free fatty acids, which contribute to lipotoxicity and are redirected into the aberrant synthesis of bioactive lipids such as ceramides and other sphingolipid species (Green et al. 2021). These structural and signaling molecules participate in key cellular processes, including proliferation, apoptosis, and mitochondrial function (Futerman 2021). Mounting evidence suggests that dysregulated sphingolipid metabolites, primarily ceramides (Cer), play a central role in the development of T2DM and other obesity‐related comorbidities, including insulin resistance and cardiovascular disease (Delcheva et al. 2024).

We hypothesized that RYGB modulates the plasma sphingolipid profile, and this effect is associated with metabolic improvement in women with obesity and T2DM. This study aimed to identify early changes in circulating sphingolipid metabolites following RYGB and to explore their metabolic associations. Specifically, we investigated correlations between sphingolipids and markers of glucose and lipid metabolism, as well as anthropometry and body composition measures, used here as a surrogate indicator of metabolic improvement.

## 2 Methods

### 2.1 Study Design and Ethical Issues

We conducted a pilot metabolomic cohort study with descriptive and associative analyses, using prospectively collected data from the SURMetaGIT study—a pan‐omic investigation registered at www.clinicaltrials.gov (NCT01251016) (Sala et al. 2016). The specific protocol for the present sphingolipid analysis was approved by the local ethics committee (CAPPesq n° 6.054.077) and adheres to the STROBE Statement for cohort studies (Appendix A in Supporting Information). All participants provided written informed consent before inclusion.

### 2.2 Casuistic

Participants from the SURMetaGIT cohort were recruited between April 2012 and March 2016 at the Bariatric and Metabolic Surgery Department of the Hospital das Clínicas, University of São Paulo Medical School. Eligibility criteria were designed to reflect the primary target population of the surgical center while minimizing sample heterogeneity and missing data. Eligible participants were biological females aged 18–60 years with grade II or III obesity (BMI 35–50 kg/m^2^) and confirmed type 2 diabetes mellitus (T2DM), as defined by fasting glucose ≥ 126 mg/dL, HbA1c ≥ 7%, or use of antidiabetic medication, who provided written informed consent. Patients were excluded if they were enrolled in other research protocols or presented with abdominal hernia, *Helicobacter pylori infection*, insulin use, or liver or thyroid dysfunction. Thirty patients met these criteria and underwent RYGB with standardized limb lengths (~50–60 cm for the biliopancreatic limb and ~100–120 cm for the alimentary limb).

### 2.3 Biochemical Markers of Glucose and Lipid Metabolism

All biochemical analyses were performed at the Central Laboratory of HC‐FMUSP at baseline and 3 months after RYGB. Fasting plasma glucose was measured by the hexokinase enzymatic method; total cholesterol and its fractions were measured using the colorimetric enzymatic CHOD/PAP (Cholesterol Oxidase/Peroxidase‐Aminoantipyrine) method; HbA1c was measured by HPLC (High‐Performance Liquid Chromatography) by Ion Exchange Chromatography—Variant II; and insulin and C‐peptide levels were assessed by electrochemiluminescence immunoassay. More information (equipment, companies, reagents, and references) about the biochemical analysis can be obtained at https://patologiaclinica.hc.fm.usp.br/lista‐manual‐exames. T2DM remission was determined based on fasting glucose and HbA1c levels, according to the 2021 American Diabetes Association (ADA) criteria (Riddle et al. 2021).

### 2.4 Body Composition and Anthropometry

Data on body composition and anthropometry were assessed at baseline and 3 months after RYGB. Body weight (kg) and height (cm) were measured using an electronic scale (Lucastec, Brazil) and a stadiometer (Sanny, American Medical of Brazil), respectively. Waist and hip circumferences were measured using a non‐elastic measuring tape and expressed in centimeters. Body mass index (BMI) was calculated as body weight (in kilograms) divided by height squared (in meters squared). Body composition was assessed using air displacement plethysmography (BOD POD; COSMED, USA) and expressed in both absolute values (kg) and percentages.

### 2.5 Circulating Sphingolipid Analyses

We analyzed data on the plasma abundance of the 32 sphingolipids identified through the untargeted metabolomic analysis of the SURMetaGIT protocol. In this protocol, plasma was obtained from blood samples collected in EDTA‐containing tubes at baseline and 3 months after RYGB, following a 12‐h overnight fast and a 4‐day interruption of antidiabetic medications. Plasma sphingolipids were extracted using a methanol‐based aqueous solution and analyzed by untargeted metabolomics using ultra‐performance liquid chromatography coupled to mass spectrometry (UPLC–MS; Agilent 6530 Accurate‐Mass Q‐TOF LC/MS and Agilent 1290 Infinity II LC System with a Charged Surface Hybrid [CSH] column; Agilent Technologies), performed at the NIH West Coast Metabolomics Center (Davis, CA, USA).

All biological samples were analyzed together, and acquisition was performed in a randomized manner. Analytical variation was controlled with quality control (QC) samples, composed of a mixture of reference molecules covering all chemical classes of metabolites identified in typical analyses (disodium EDTA plasma—HMPLEDTA, industrially produced by Bioreclamation IVT—Westbury, NY/USA), and isotope‐modified internal standards to adjust the sample scale with library conditions through retention time correction were also used. QC samples were systematically interspersed every 10 patient samples throughout the analytical sequence to monitor analytical conditions (signal intensity, retention time, and stability of assays performed over time) and to evaluate technical variations that could compromise analysis quality. In this process, metabolite peaks are detected (peak picking), retention times are aligned, and then the relative quantity of metabolites, or abundance, is measured.

The peak intensities of the metabolites were determined by mass spectrometry with a spectrometer configured to measure a mass‐to‐charge ratio (m/z) scan range between 60 and 1700 with a speed of 2 spectra per second, under a constant flow of 6.0 mL/min and an injection volume of 1.67 and 5 μL for the positive and negative modes, respectively. The entire acquisition was performed with the samples stored at 4°C. Changes in the ratio of peak heights and the concentration of analytes were monitored.

The identification of metabolites was based on comparing the accurate mass‐to‐charge ratio (m/z) and retention time data with the corresponding spectra in the West Coast Metabolomics Center libraries. The plasma data were normalized for subsequent analyses by the sum of the intensity of all identified metabolites (mTIC). After normalization, metabolites with a coefficient of variation of less than 20% in the QC samples were considered viable metabolite data. Metabolites measured in < 50% of the samples, which could not be separated based on chromatographic elution patterns and division into m/z ionic spectra, as well as those that were confused with potential contaminants, were excluded. In addition, when sphingolipids were identified in both positive and negative ionization modes, the metabolite with the higher CV was excluded.

Data were processed and analyzed using MS‐DIAL software (http://prime.psc.riken.jp/Metabolomics_Software/). Sphingolipids were identified and annotated using the online tools CTS Fiehn Lab and LIPID MAPS, respectively. A detailed description of sample preparation, instrument configuration, and metabolite identification of sphingolipids from the SURMetaGIT study can be found in Appendix B in Supporting Information.

### 2.6 Sample Size and Statistical Analysis

The original sample size (*n* = 20) of the SURMetaGIT cohort was estimated based on transcriptomic outcomes, with 10 additional participants further included for gene expression validation. Thus, the present study uses a convenience sample from the original cohort for sphingolipid analysis. A post hoc power analysis was performed using the SSPA R package available in MetaboAnalyst 6.0, considering 32 identified sphingolipids, a paired study design, and a false discovery rate (FDR) threshold of 0.03. Under these parameters, the effective sample size (*n* = 28) yielded an estimated statistical power exceeding 90%.

Inferential statistical analysis was conducted using Jamovi software version 2.2.5. Paired *t*‐tests were used for pre‐ and postoperative comparisons, with data normality verified by the Shapiro–Wilk test. Effect sizes were calculated using Cohen's *d*, expressing the magnitude of metabolite changes after surgery.

Exploratory analysis of sphingolipids was performed using MetaboAnalyst versions 5.0 and 6.0, including partial least squares discriminant analysis (PLS‐DA), volcano plots, and heatmaps. For the volcano plot, data were autoscaled, and *p* values were adjusted using the FDR method to control for multiple comparisons across the 32 sphingolipids (Appendix C in Supporting Information). The fold change cutoff was ±1.2, defined considering the paired study design and limited sample size (Psychogios 2009). Fold change was calculated as log_2_ (postoperative mean/preoperative mean). A 5% significance level was adopted.

Generalized Estimating Equations (GEE) were also applied to absolute sphingolipid values to assess differences between time points, controlling for confounding variables such as age and BMI. Time (pre‐ vs. post‐surgery) was the primary predictor, and Gaussian or gamma distributions were employed based on the symmetry of the data. Results were expressed as estimated coefficients (β) and corresponding *p*‐values (Appendix D in Supporting Information).

Finally, sphingolipid levels were correlated with biochemical markers of glucose and lipid metabolism, as well as with anthropometric and body composition data, used as surrogate markers of metabolic improvement. Correlation analyses were performed using Spearman's rank test, with a significance threshold of *p* < 0.05.

## 3 Results

### 3.1 Descriptive Data

Thirty patients were included. At baseline, they had a mean age of 47.3 years and a mean BMI of 45.5 kg/m^2^, with marked central adiposity and poor glycemic and lipid control, evidenced by a mean fasting glucose of 215.8 mg/dL, a mean HbA1c of 8.9%, elevated LDL‐c and triglyceride levels, and low HDL‐c (Table 1).

**TABLE 1:** Baseline characteristics of study participants (*n* = 30).

Variable	Mean ± SD
Age (years)	47.3 ± 6.9
Height (m)	1.6 ± 0.1
Weight (kg)	113.8 ± 15.1
BMI (kg/m^2^)	45.5 ± 5.7
Fat (%)	51.8 ± 6.4
Fat (kg)	58.9 ± 13.5
Lean mass (%)	48.2 ± 6.4
Lean mass (kg)	53.9 ± 7.0
Arm circumference (cm)	44.7 ± 14.7
Waist circumference (cm)	127.1 ± 12.3
Hip circumference (cm)	136.9 ± 12.4
Waist‐hip ratio	0.93 ± 0.08
Glucose (mg/dL)	215.8 ± 72.4
Insulin (μU/mL)^a^	21.6 ± 14.7
HbA1c (%)	8.9 ± 1.6
C‐peptide (ng/mL)	4.0 ± 1.3
Total cholesterol (mg/dL)	193.7 ± 35.6
HDL‐c (mg/dL)	43.7 ± 9.8
Non‐HDL‐c (mg/dL)	149.9 ± 33.7
LDL‐c (mg/dL)	117.3 ± 33.9
VLDL‐c (mg/dL)	29.9 ± 9.5
Triglycerides (mg/dL)^a^	169.0 ± 90.6

Abbreviations: BMI, body mass index; HbA1c, glycated hemoglobin; HDL‐c, high‐density lipoprotein cholesterol; IQR, interquartile range; LDL‐c, low‐density lipoprotein cholesterol; SD, standard deviation; VLDL‐c, very‐low‐density lipoprotein cholestero.

^a^ Insulin and triglyceride levels were not normally distributed and are also reported as medians with interquartile ranges (IQR): Insulin = 16.5 μU/mL (IQR: 13.45–25.1); Triglycerides = 150 mg/dL (IQR: 108.75–203).

### 3.2 Postoperative Metabolic Improvements

All patients showed improvement in all evaluated parameters of glucose metabolism: fasting plasma glucose (215.8 ± 72.4 vs. 104 ± 21.7 mg/dL), insulin (median 16.5 ± 11.0 vs. 7.9 ± 6.1 μUI/mL), HbA1c (8.9% ± 1.6% vs. 6.04% ± 0.5%), and C‐peptide (4.02 ± 1.3 vs. 2.9 ± 0.8 ⴄUI/mL), respectively—*p* < 0.001. Moreover, 3 months after surgery, 18 patients (64.3%) achieved incident remission of T2DM, while the remaining participants showed a substantial reduction in their use of oral antidiabetic medications.

Regarding lipid metabolism, a significant reduction in total cholesterol (193.7 ± 35.6 vs. 162.3 ± 48.4 mg/dL, *p* < 0.001) was observed, primarily driven by decreases in pro‐atherogenic fractions: non‐HDL‐c (149.9 ± 33.7 vs. 112.2 ± 52.9 mg/dL, *p* < 0.001), LDL‐c (117.3 ± 33.9 vs. 97.2 ± 37.8 mg/dL, *p* = 0.01), VLDL‐c (29.9 ± 9.5 vs. 23 ± 9.3 mg/dL, *p* = 0.002), triglycerides (median 150 ± 85.2 vs. 104.5 ± 36.7 mg/dL, *p* = 0.004). HDL‐c showed no significant changes (43.7 ± 9.8 vs. 42.1 ± 9.4 mg/dL, *p* = 0.185).

**FIGURE 1:**
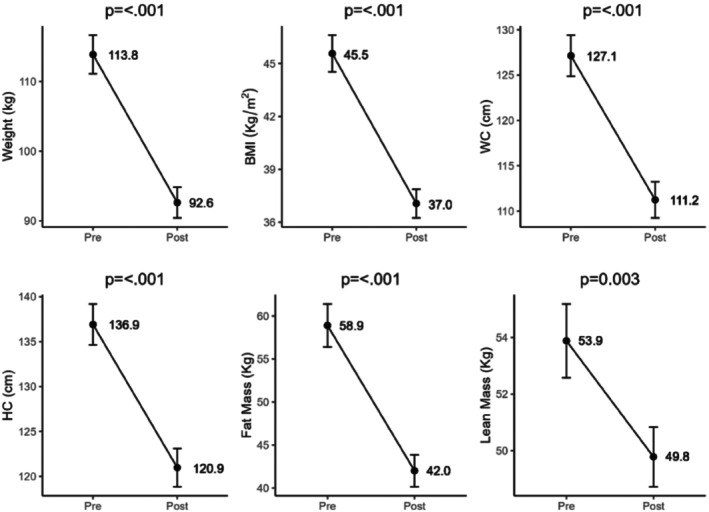
Improvements in body composition and anthropometric measures 3 months after Roux‐en‐Y gastric bypass. This graph illustrates the changes in mean values between pre‐ and postoperative periods. The *p* value expresses the significant differences between the post‐ and preoperative periods (*t*‐test). BMI, body mass index; HC, hip circumference; WC, waist circumference.

In parallel, significant reductions in body weight and BMI, along with improvements in body composition, were also observed (Figure 1). The mean percentage of weight loss at 3 months was 18.4%.

### 3.3 Sphingolipid Remodeling

Due to postoperative edema, two participants were unable to provide sufficient plasma for sphingolipid assessment, resulting in a final sample of 28 individuals for this analysis. A total of 32 sphingolipids were identified and retained for analysis. The sphingoid base composition was as follows: d16:1 sphingosine (3.1%), d18:0 sphinganine (12.5%), d18:1 sphingosine (62.5%), and d18:2 sphingadiene (21.9%).

**FIGURE 2:**
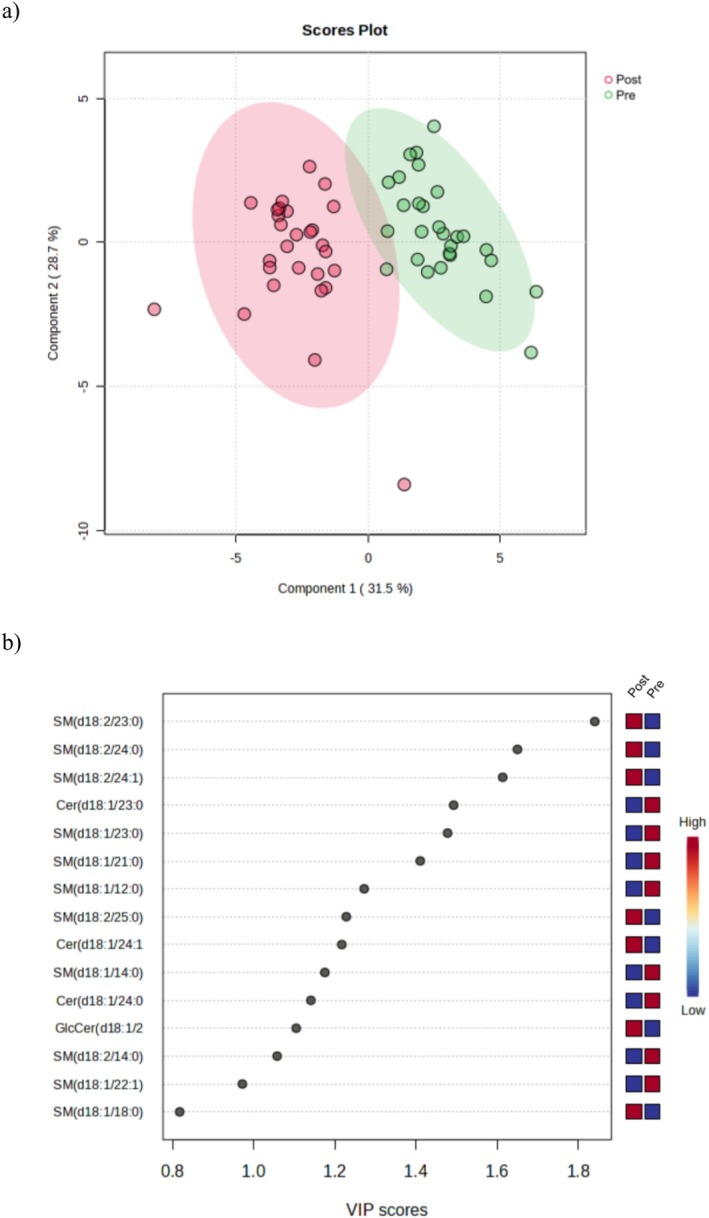
Metabolites dimensionality in the pre‐ and post‐operative periods. (a) Partial Least Squares Discriminant Analysis (PLS‐DA); The PLS‐DA model is a form of supervised dimensionality reduction. It assigns groupings to each sample (in this case, pre‐ and post‐intervention) by creating new latent variables called components. In this figure (a), a satisfactory clustering between the groups can be observed, highlighting the pre‐ and post‐intervention conditions, suggesting that the model was able to differentiate the two groups based on the created components, particularly Component 1, which accounts for 31.5% of the initial variance in the metabolic data. (b) Variable Importance in Projection (VIP) scores are values that quantify the weight of each variable in a model's construction, highlighting the most discriminant sphingolipids between time points. The top 15 VIP scores in the model, as shown in this figure, indicate that a higher VIP value means greater importance for the model. Conventionally, a VIP score of 1 or greater is considered an important variable. Red color means higher absolute abundance, whereas blue color means lower absolute abundance.

Postoperative changes in sphingolipid profiles revealed a clear distinction from the preoperative state (Figure 2), with 21 of the 32 sphingolipids showing statistically significant alterations (*q* < 0.05).

**FIGURE 3:**
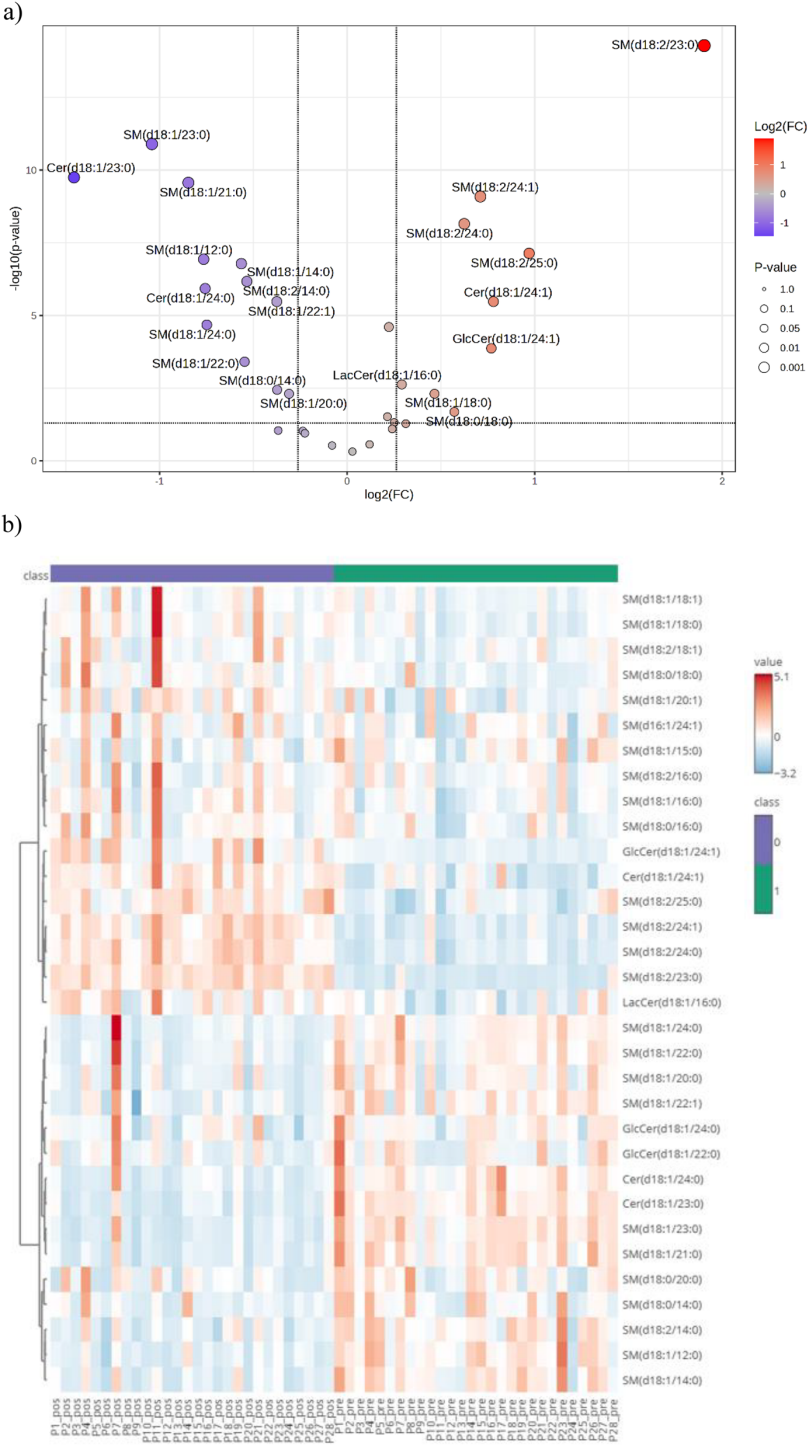
Changes in the magnitude and distribution patterns of sphingolipids before and after Roux‐en‐Y gastric bypass. (a) Volcano plot: this scattered plot is used in large data sets to identify changes between two time points quickly. For this analysis, the data were previously normalized using the autoscaling method (mean centering and scaling by variance), followed by the correction of *p* values using the False Discovery Rate (FDR) method. The cutoff for the log_2_ fold change (FC) was set at ±1.2, and metabolites with an adjusted *p* value < 0.05 were considered statistically significant. Metabolites that were significantly increased after surgery are highlighted in red, and those that were reduced are in blue. (b) Heatmap—this is a bidimensional data visualization technique that expresses the magnitude of individual values within a dataset in terms of color, typically using red or blue. The map shows relative abundance across participants before (right side, under the green box) and 3 months after (left side, under the purple box) surgery. The red color means increased relative metabolite abundance, whereas the blue color means decreased relative abundance.

Sphingolipids containing unsaturated fatty acyl chains had an overall increase. These changes are expressed in the volcano plot and heatmap (Figure 3). In contrast, those with saturated chains attached to d18:1 sphingosine decreased, except for the species with 16–18 carbons in the fatty acyl chain. Long‐chain sphingomyelins (23–25 carbons in the fatty acyl chain) linked to d18:2 increased, whereas those with 14 carbons in the fatty acyl chain decreased. Similarly, sphingomyelin species containing 16–18 carbons in the fatty acyl chain linked to d18:0 sphinganine increased, but those containing 14 side‐chain carbons again decreased. Neither age nor BMI significantly influenced metabolite levels, reinforcing that the observed changes were primarily associated with the surgical time point (Appendix D in Supporting Information).

In correlation analyses, most sphingolipids showed weak associations with glycemic markers. Among individuals who achieved T2DM remission, SM(d18:0/14:0) and SM(d18:2/25:0) exhibited significant postoperative changes—SM(d18:0/14:0) decreased (Cohen's *d* = 0.61) and SM(d18:2/25:0) increased (Cohen's *d* = 0.99)—but neither species correlated with glycemic parameters, suggesting the influence of other mechanisms or statistical chance. In contrast, stronger associations were observed with lipid profile, particularly LDL‐c, after RYGB. Notably, Cer(d18:1/24:0) correlated strongly with total cholesterol (*r* = 0.84), while SM(d18:1/20:0) and SM(d18:1/22:0) showed high correlations with both total cholesterol (*r* = 0.88 and r = 0.83, respectively) and LDL‐c (*r* = 0.78 and 0.76). Additional positive correlations with LDL‐c were identified for SM(d18:1/21:0) (*r* = 0.71) and SM(d18:2/23:0) (*r* = 0.76). The full set of correlations between sphingolipids and biochemical markers of lipid homeostasis is presented in Table 2. Correlation analysis of sphingolipids with glycemic markers is expressed in Appendix E in Supporting Information.

**TABLE 2:** Correlation analysis between plasma sphingolipids and lipid profile markers before and after Roux‐en‐Y gastric bypass.

	Total cholesterol mg/dL	HDL‐c mg/dL	Non‐HDL‐c mg/dL	LDL‐c mg/dL	VLDL‐c mg/dL	Triglycerides mg/dL
Pre‐surgery (*n* = 28)						
Cer(d18:1/23:0)	0.67***	0.09	0.60***	0.44*	0.09	0.27
Cer(d18:1/24:0)	0.63***	−0.07	0.65***	0.44*	0.23	0.38*
Cer(d18:1/24:1)	0.10	−0.44*	0.24	0.13	0.42*	0.53**
GlcCer(d18:1/24:1)	0.50**	0.03	0.53**	0.32	0.36	0.33
SM(d18:1/12:0)	0.17	0.03	0.18	0.21	−0.06	−0.09
SM(d18:0/14:0)	0.46*	0.05	0.43*	0.30	0.04	0.12
SM(d18:1/14:0)	0.51**	0.22	0.44*	0.44*	−0.05	0.00
SM(d18:2/14:0)	0.09	0.34	−0.07	−0.05	−0.21	−0.23
SM(d18:1/18:0)	0.38*	0.25	0.29	0.24	0.02	0.06
SM(d18:1/20:0)	0.53**	0.33	0.43*	0.35	0.00	0.09
SM(d18:1/21:0)	0.56**	0.26	0.47*	0.41*	−0.06	0.06
SM(d18:1/22:0)	0.49**	0.12	0.46*	0.40*	−0.06	0.11
SM(d18:1/22:1)	0.16	0.38*	0.00	0.06	−0.48*	−0.47*
SM(d18:1/23:0)	0.51**	0.29	0.42*	0.37	−0.11	0.03
SM(d18:1/24:0)	0.48*	0.07	0.49**	0.31	0.06	0.18
SM(d18:2/24:1)	−0.19	−0.02	−0.20	−0.08	−0.37	−0.44*
Post‐surgery (*n* = 28)						
Cer(d18:1/23:0)	0.79***	0.32	0.59***	0.69***	0.61***	0.59**
Cer(d18:1/24:0)	0.84***	0.45*	0.57**	0.70***	0.54**	0.52**
Cer(d18:1/24:1)	0.68***	0.12	0.51**	0.67***	0.71***	0.69***
GlcCer(d18:1/24:1)	0.45*	0.42*	0.25	0.42*	0.11	0.11
SM(d18:1/12:0)	0.43*	0.25	0.45*	0.41*	0.38	0.37
SM(d18:0/14:0)	0.49*	0.02	0.41*	0.54**	0.18	0.18
SM(d18:1/14:0)	0.71***	0.41*	0.49**	0.64***	0.48*	0.47*
SM(d18:2/14:0)	0.60**	0.45*	0.45*	0.55**	0.27	0.26
SM(d18:0/18:0)	0.03	0.16	−0.11	0.01	−0.21	−0.22
SM(d18:1/18:0)	0.56**	0.39*	0.40*	0.54**	0.39	0.37
SM(d18:1/20:0)	0.88***	0.51**	0.58**	0.78***	0.46*	0.44*
SM(d18:1/21:0)	0.79***	0.41*	0.63***	0.71***	0.49*	0.47*
SM(d18:1/22:0)	0.83***	0.53**	0.51**	0.76***	0.38	0.36
SM(d18:1/22:1)	0.58**	0.50**	0.40*	0.51**	−0.01	−0.02
SM(d18:1/23:0)	0.71***	0.46*	0.52**	0.63***	0.35	0.33
SM(d18:2/23:0)	0.73***	0.25	0.56**	0.76***	0.51**	0.50**
SM(d18:1/24:0)	0.69***	0.56**	0.36	0.60**	0.21	0.19
SM(d18:2/24:0)	0.38	0.31	0.23	0.44*	0.10	0.08
SM(d18:2/24:1)	0.16	0.08	0.12	0.27	−0.17	−0.18

*Note:* This table shows Spearman‘s correlation coefficient of sphingolipids analyzed and plasma lipid markers. Negative correlations are presented with minus sign (−) in red color, and positive correlations are expressed in blue color. The more intense colors in the boxes represent stronger correlations. *p < 0.05, **p < 0.01, *p* < 0.001.

Regarding body composition, only two ceramides—Cer(d18:1/23:0) and Cer(d18:1/24:0)—showed moderate associations. Before surgery, these ceramides showed a negative correlation with the fat mass percentage (*r* = 0.51 and 0.58, respectively) and a positive correlation with the lean mass percentage (*r* = 0.51 and 0.58, respectively). Additionally, after surgery, Cer containing 23 and 24 carbons in the fatty acyl chain showed negative correlations with fat mass percentage (*r* = 0.45 and 0.46) and positive correlations with lean mass percentage (*r* = 0.45 and 0.46).

## 4 Discussion

RYGB reduces plasma fatty acid precursors involved in de novo sphingolipid synthesis (Machado, 2018). Furthermore, activation of TLR4 is diminished (Sala et al. 2020). These molecular alterations are anticipated to reduce sphingolipid synthesis after surgery. However, previous studies have reported both decreases and increases in saturated sphingolipids following RYGB, suggesting that additional mechanisms—possibly involving the intestinal microbiota—may regulate sphingolipid remodeling. Nonetheless, the complex molecular pathways underlying these changes remain to be fully elucidated. Overall, an increase in unsaturated ceramides and sphingomyelins, and a decrease in polyunsaturated sphingomyelins, can be expected (Kayser et al. 2017; Fiamoncini et al. 2018; Balonov et al. 2023).

The lack of a consistent pattern in sphingolipid alterations raises questions about the precise roles of individual sphingolipid species in metabolic pathways. This complexity underscores the need to investigate how these lipid species differentially contribute to cellular functions and systemic metabolism. For instance, Endapally et al. demonstrated that specific sphingolipids exhibit distinct conformational structures within the plasma membrane, which can modulate their interactions with sensor proteins and affect binding affinities (Endapally et al. 2019). If plasma sphingolipid remodeling reflects cellular lipid rearrangements in the plasma membrane, it is plausible that these changes play a role in metabolic improvement following metabolic and bariatric surgery. These lipid dynamics could enhance signal transduction processes critical for metabolic regulation by influencing the interactions between sensor proteins and their ligands. Future studies are warranted to explore these mechanisms and delineate the specific contributions of sphingolipid species to post‐surgical metabolic outcomes.

In our study, the overall levels of saturated ceramides and sphingomyelins decreased, except for sphingomyelins with 16 and 18 carbons in the fatty acyl chain. We identified four sphingoid bases within sphingolipids: d16:1 sphingosine, d18:0 sphinganine, d18:1 sphingosine, and d18:2 sphingadiene. As expected, D‐erythro‐sphingosine accounted for most of the sphingoid bases in plasma sphingolipids. Distinct sphingoid bases may influence sphingolipid remodeling; however, this finding requires further exploration.

Malabsorptive procedures also present contrasting findings regarding post‐operative sphingolipid remodeling. Biliopancreatic diversion has been associated with decreased sphingomyelins and sphingolipids (Ramos‐Molina et al. 2018), whereas RYGB is associated with increased sphingomyelins (Samczuk et al. 2018). It is worth noting that changes in sphingomyelin levels after RYGB were previously linked to caloric restriction rather than the surgical procedure itself (Herzog et al. 2020).

In this protocol, we found only a moderate and consistent correlation between sphingolipids and body composition before and after surgery, despite the known relationship between obesity, lipotoxicity, and sphingolipids. Body fat percentage was negatively correlated with ceramides with 23 and 24 carbons in the fatty acyl chain. Conversely, lean mass percentage was positively correlated with these ceramides.

Circulating ceramides have been consistently associated with increased cardiovascular risk (Havulinna et al. 2016). Moreover, some ceramides have been shown to predict cardiovascular disease independently of cholesterol, such as 16:0, 18:0, 24:0, and 24:1 ceramides included in the CERT1 score (Hilvo et al. 2020). The plasma Cer(d18:1/24:1)‐to‐Cer(d18:1/24:0) ratio, part of CERT1, is consistently associated with higher cardiovascular risk in several studies, notably higher levels of Cer(d18:1/24:1) (Cao et al. 2020; Laaksonen et al. 2016; Mantovani and Dugo 2020). In our study, the Cer(d18:1/24:1)‐to‐Cer(d18:1/24:0) ratio increased from 0.28 to 0.79 post‐surgery, and Cer(d18:1/24:1) showed an increased fold change with a large effect size (0.96), contradicting the expected improvement in this biomarker following RYGB, given the proven cardiovascular benefits of metabolic and bariatric surgery (van Veldhuisen et al. 2022). Nevertheless, the short‐term follow‐up must be emphasized, and the other ceramides included in the CERT1 score—16:0 and 18:0—were not identified in this untargeted analysis.

Regarding glycemic metabolism, impaired sphingolipid metabolism and increased ceramide levels have been associated with insulin resistance, T2DM, and other metabolic diseases (Boini et al. 2018), particularly high serum levels of 16:0 ceramide derived from palmitic acid (Hilvo et al. 2020). This ceramide was not identified in our analysis, limiting our ability to assess its role in glycemic improvement and T2DM remission after surgery. Additionally, we did not observe sustained or strong correlations between sphingolipids and fasting glucose, insulin, C‐peptide, or HbA1c, contrasting with the findings of Poss et al. (2022). who reported that lower post‐surgical ceramides with 16–24 carbons in the fatty acyl chain and their baseline levels predicted T2DM remission.

Regarding lipid metabolism, we found that sphingolipids showed a positive correlation with total cholesterol and LDL‐c, especially after surgery, with strong correlations (*ρ* > 0.8) observed with long‐chain saturated sphingolipids, such as 24:0 Cer, SM(d18:1/20:0), and SM(d18:1/22:0). However, correlations with HDL‐c and other cholesterol fractions were not as prominent. This finding is consistent with the report by Kayser et al. (2017) and is biologically plausible, given the essential role of sphingolipids in lipid raft formation alongside cholesterol (Jiang and Li 2022). Inhibition of ceramide or sphingomyelin synthesis has been associated with reduced plasma cholesterol levels (Park et al. 2006). Specifically, a decrease in sphingomyelin content in the plasma membrane leads to lower plasma cholesterol (Gupta and Rudney 1991). Finally, administration of myriocin—an SPT inhibitor—to apolipoprotein E knockout mice has been shown to reduce serum lipids and cardiovascular disease markers, mitigating atherosclerosis progression (Hojjati et al. 2005; Park et al. 2008).

Given the dynamic nature of sphingolipid metabolism, our findings should be interpreted with caution. The observed short‐term alterations may reflect an adaptive response or a transient phase in the progression toward metabolic homeostasis. Distinct sphingolipid profiles reported in long‐term follow‐up studies further highlight the potential for temporal variability influenced by both intrinsic and extrinsic factors (Samczuk et al. 2018; Poss et al. 2022). Environmental variables such as diet, gut microbiota composition, and physical activity likely contribute to these shifts in sphingolipid profiles. Alternatively, the observed changes may represent a gradual transition toward a new metabolic equilibrium, in which the sphingolipid profile reflects the evolving metabolic state rather than serving as the primary driver of change. Importantly, these possibilities are not mutually exclusive, emphasizing the need for integrative approaches that combine longitudinal cohort studies with mechanistic research to elucidate the interplay between sphingolipid dynamics and metabolic outcomes.

### 4.1 Study Limitations

This study has some limitations that warrant cautious interpretation, including its single‐center design, small sample size, short‐term follow‐up, and inclusion of only women without specific comorbidities. These factors may limit the generalizability of our findings and reduce the power to detect subtler metabolic alterations. Nonetheless, the homogeneity of the cohort enhances the internal validity of our observations, particularly the associations between specific sphingolipid species and cholesterol metabolism following RYGB.

## 5 Conclusion

Sphingolipids are bioactive lipids involved in the pathophysiology of metabolic diseases and exhibited selective changes after RYGB in this exploratory study. In our sample, surgically induced alterations in sphingolipids were more strongly associated with cholesterol levels, particularly LDL‐c, than with glycemic markers or body composition. SM(d18:1/20:0) and SM(d18:1/22:0) showed the strongest correlations with both total cholesterol and LDL‐c after surgery. These findings point to a potential role of specific sphingolipid species in the plasma lipid response to RYGB. Further prospective studies with larger cohorts are needed to confirm these associations and clarify their clinical relevance.

## Author Contributions

Gabriela de Oliveira Lemos contributed to the study's conceptualization and design, as well as the development of data analysis tools, and wrote the original draft. Raquel Susana Torrinhas contributed to study design and data analysis, supervised the project, and critically reviewed the manuscript. Natasha Mendonça Machado contributed to study design and analysis tools, data collection and curation, supervised the project, and critically reviewed the manuscript. Dan Linetzky Waitzberg conceived and designed the study, acquired funding, administered and supervised the project, and critically reviewed the manuscript.

## Ethics Statement

This study was conducted in accordance with the Declaration of Helsinki and was approved by the local ethics committee (Comissão de Ética para Análise de Projetos de Pesquisa—CAPPesq), with approval number 6.054.077 (approval date: May 11, 2023).

## Consent

Informed consent was obtained from all subjects involved in the study.

## Conflicts of Interest

The authors declare no conflicts of interest.

## Supporting information


**Appendix A** STROBE statement on cohort studies.

**Appendix B** SURMetaGIT LC–MS procedures.

**Appendix C** False discovery rate (FDR) score.

**Appendix D** Generalized estimating equations (GEE) model.

**Appendix E** Correlation analysis of sphingolipids with glycemic markers.

## Data Availability

The data that support the findings of this study are available on request from the corresponding author. The data are not publicly available due to privacy or ethical restrictions.
