# Supplementary material for: Changes in Plasma Sphingolipid Metabolites Following Roux‐En‐Y Gastric Bypass in Women With Obesity and Type 2 Diabetes: A Pilot Metabolomic Cohort Study
Source: Lipids. 2025 Nov 12;61(2):195–205. doi: 10.1002/lipd.70019 (PMC12975409; doi:10.1002/lipd.70019)
Supplement: Supplementary file 2 — Appendix B SURMetaGIT LC–MS procedures. [file LIPD-61-195-s003.pdf]

Appendix B. SURMetaGIT LC-MS procedures

a) Sample Preparation Procedures:

LC-MS *Metabolite Extraction*

1. Thaw the sample aliquot on ice.
2. Add 225µL of ice-cold N_2-purified MeOH + QC mix.
3. Vortex for 10s.
4. Add 750µL of ice-cold methyl tert-butyl ether (MTBE) with 22:1 cholesterol ester (CE).
5. Vortex for 10s.
6. Add 200µL of miliQ water.
7. Homogenize in an orbital shaker for 6 min at 4°C.
8. Vortex for 20s and centrifuge for 2 min at 1400 rcf.
9. Transfer the supernatant (apolar phase) to micro-eppendorf tubes (300µL).
10. Dry the samples with apolar (for CSH) extractions in a speedvac.
11. Store the remaining tubes (backup) at -20°C.

Reconstitution for LC-MS (CSH):

1. Add 110µL of MeOH:Tol 1:10 + 50mg/mL of 12-[[cyclohexylamino)carbonyl]amino-dodecanoic acid (CUDA).
2. Vortex for 20s.
3. Sonicate for 5 min at room temperature.
4. Centrifuge for 2 min at 1400 rcf.
5. Transfer 100µL to amber glass microtubes with microsert.

---

b) Instrument Configuration

Instrument 1 – Liquid Chromatography coupled with Mass Spectrometer (LC-MS) Agilent 1290 Infinity II LC System Agilent 6530 Accurate-Mass Q-TOF LC/MS

Acquisition Mode Analyses were performed with a flow rate of 6.0 mL/min and an injection volume of 1.67 µL for positive mode and 5 µL for negative mode. The instrument was configured to measure molecules with a scan range between 60 and 1,700 m/z and a speed of 2 spectra per second. Electrospray ionization was applied at +305kV. During the measurement of apolar molecules with the CSH column, MS/MS fragmentation was performed with a collision energy of 20eV for both positive and negative modes. The samples were kept at 4°C during the entire acquisition. More details are described below:

Mobile Phase: 400µL/min (injection flow)

- A: 60% acetonitrile in ultrapure water
- B: 100% isopropanol Gradient: 0.6 µL/min
- 0 - 2 min: 70% A and 30% B
- 2 - 2.5 min: 52% A and 48% B
- 2.5 - 11 min: 18% A and 82% B
- 11 - 12 min: 1% A and 99% B
- 12.10 to 15 min: 85% A and 15% B

C18 Stationary Phase: ACQUITY UPLC® CSH™ C18 column (Waters-Part n° 186005297) 2.1X150mm, 1.7µm particle size.

---

c) Metabolite Identification

Metabolite identification was based on accurate mass and accurate retention time, which were compared with the WCMC libraries containing corresponding spectra. All BinBase entries were compared with mass spectra from the FiehnLib library, which contains 1,200 authentic spectra using retention index and mass spectra information, or with library 11 from the National Institute of Standards and Technology (NIST). Metabolites with a coefficient of variation of <20% in the QC samples after normalization were reported in subsequent analyses. The following metabolites were excluded:

1. Those that were mistaken for potential contaminants;
2. Those that were measured in <50% of the samples;
3. Those that could not be separated based on the chromatographic elution patterns and division into "parent and daughter" m/z ionic spectra.

Composition of the QC mix for CSH UPLC/MS: *Analyte Name, Formula, MS1 m/z, RT (min)*

- CE (22:1) [M+Na]+ iSTD, $C_{49}H_{86}O_2$, 729.652, 11.727
- CE (22:1) [$M+NH_4$]+ iSTD, $C_{49}H_{86}O_2$, 724.6966, 11.727
- Ceramide C17 [M+H]+ iSTD, $C_{35}H_{69}NO_3$, 552.535, 5.948
- Ceramide C17 [$M+H-H_2O$]+ iSTD, $C_{35}H_{69}NO_3$, 534.5245, 5.948
- Ceramide C17 [M+Na]+ iSTD, $C_{35}H_{69}NO_3$, 574.517, 5.948
- Cholesterol d7 [$M-H_2O+H$]+ iSTD, $C_{27}H_{39}D_7O$, 376.3955, 4.787
- CUDA (pos) iSTD [M+H]+, $C_{19}H_{36}N_2O_3$, 341.2799, 0.774
- DG (12:0/12:0/0:0) [M+Na]+ iSTD, $C_{27}H_{52}O_5$, 479.3707, 4.248

- DG (12:0/12:0/0:0) [M+NH4]+ iSTD, $C_{27}H_{52}O_5$, 474.4153, 4.248
- DG (18:1/2:0/0:0) [M+Na]+ iSTD, $C_{23}H_{42}O_5$, 421.2925, 3.162
- DG (18:1/2:0/0:0) [M+NH4]+ iSTD, $C_{23}H_{42}O_5$, 416.3371, 3.162
- LPC (17:0) [M+H]+ iSTD, $C_{25}H_{52}NO_7P$, 510.3554, 1.827
- LPE (17:1) [M+H]+ iSTD, $C_{22}H_{44}NO_7P$, 466.2928, 1.346
- MG (17:0/0:0/0:0) [M+H]+ iSTD, $C_{20}H_{40}O_4$, 345.2999, 3.038
- MG (17:0/0:0/0:0) [M+Na]+ iSTD, $C_{20}H_{40}O_4$, 367.2819, 3.038
- MG (17:0/0:0/0:0) [M+NH4]+ iSTD, $C_{20}H_{40}O_4$, 362.3265, 3.038
- PC (12:0/13:0) [M+H]+ iSTD, $C_{33}H_{66}NO_8P$, 636.4596, 3.502
- PE (17:0/17:0) [M+H]+ iSTD, $C_{39}H_{78}NO_8P$, 720.5538, 6.263
- SM (17:0) [M+H]+ iSTD, $C_{40}H_{81}N_2O_6P$, 717.5915, 5.053
- Sphingosine (d17:1) [M+H]+ iSTD, $C_{17}H_{35}NO_2$, 286.2741, 1.04
- TG (17:0/17:1/17:0) [M+Na]+ d5 iSTD, $C_{54}H_{97}D_5O_6$, 874.7877, 10.997
- TG (17:0/17:1/17:0) [M+NH4]+ d5 iSTD, $C_{54}H_{97}D_5O_6$, 869.8323, 11.006
- CUDA: (12-[[cyclohexylamino)carbonyl]amino-dodecanoic acid) from Cayman Chemical Item, number 10007923.
