# Supplementary material for: Changes in Plasma Sphingolipid Metabolites Following Roux‐En‐Y Gastric Bypass in Women With Obesity and Type 2 Diabetes: A Pilot Metabolomic Cohort Study
Source: Lipids. 2025 Nov 12;61(2):195–205. doi: 10.1002/lipd.70019 (PMC12975409; doi:10.1002/lipd.70019)
Supplement: Supplementary file 3 — Appendix C False discovery rate (FDR) score. [file LIPD-61-195-s001.pdf]

Appendix C. Plasma metabolites false discovery rate for sphingolipids with statistical significance changes

Metabolite	$log_2(FC)$	Adjusted *p*-value
SM(d18:2/23:0)	1.9037	**<.001**
SM(d18:1/23:0)	-1.041	**<.001**
Cer(d18:1/23:0)	-1.4565	**<.001**
SM(d18:1/21:0)	-0.84674	**<.001**
SM(d18:2/24:1)	0.71041	**<.001**
SM(d18:2/24:0)	0.62422	**<.001**
SM(d18:2/25:0)	0.97019	**<.001**
SM(d18:1/12:0)	-0.76508	**<.001**
SM(d18:1/14:0)	-0.56421	**<.001**
SM(d18:2/14:0)	-0.53474	**<.001**
Cer(d18:1/24:0)	-0.75695	**<.001**
Cer(d18:1/24:1)	0.78012	**<.001**
SM(d18:1/22:1)	-0.37508	**<.001**
SM(d18:1/24:0)	-0.74825	**<.001**
GlcCer(d18:1/24:1)	0.76873	**<.001**
SM(d18:1/22:0)	-0.54683	**<.001**
LacCer(d18:1/16:0)	0.29136	**0.002**
SM(d18:0/14:0)	-0.3733	**0.003**
SM(d18:1/18:0)	0.4658	**0.004**
SM(d18:1/20:0)	-0.30966	**0.004**
SM(d18:0/18:0)	0.57144	**0.02**

The fold change was calculated as log_2 (postoperative mean / preoperative mean). A 5% significance level was adopted for adjusted p-values. In turn, p-adjusted values were calculated according to the False Discovery Rate (FDR) correction using the Benjamini-Hochberg procedure to all analyses. The minus signal (-) refers to the decrease in the fold change metabolite's abundance
