# Supplementary material for: Changes in Plasma Sphingolipid Metabolites Following Roux‐En‐Y Gastric Bypass in Women With Obesity and Type 2 Diabetes: A Pilot Metabolomic Cohort Study
Source: Lipids. 2025 Nov 12;61(2):195–205. doi: 10.1002/lipd.70019 (PMC12975409; doi:10.1002/lipd.70019)
Supplement: Supplementary file 4 — Appendix D Generalized estimating equations (GEE) model. [file LIPD-61-195-s002.pdf]

Appendix D. Generalized Estimating Equation (GEE) model for potential outcome predictors

Metabolite	Predictor Variable	β coefficient	*p*-value	*q*-value
	Time	-200	**<.001**	
SM(d18:2/23:0)	Age	0.99	0.43	**<.001**
	BMI	-0.08	0.95	
	Time	599	**<.001**	
SM(d18:1/23:0)	Age	4.84	0.45	**<.001**
	BMI	-11.5	0.14	
	Time	127	**<.001**	
Cer(d18:1/23:0)	Age	-0.61	0.55	**<.001**
	BMI	-2.45	0.16	
	Time	171	**<.001**	
SM(d18:1/21:0)	Age	0.4	0.84	**<.001**
	BMI	-3.63	0.16	
	Time	-297	**<.001**	
SM(d18:2/24:1)	Age	1.9	0.43	**<.001**
	BMI	3.65	0.25	
	Time	-501.3	**<.001**	
SM(d18:2/24:0)	Age	4.75	0.19	**<.001**
	BMI	1.03	0.83	
	Time	-56.5	**<.001**	
SM(d18:2/25:0)	Age	-0.06	0.94	**<.001**
	BMI	0.77	0.48	
	Time	12	**<.001**	
SM(d18:1/12:0)	Age	0.11	0.48	**<.001**
	BMI	-0.23	0.28	
	Time	327	**<.001**	
SM(d18:1/14:0)	Age	3.84	0.4	**<.001**
	BMI	-10.4	0.07	
	Time	25	**<.001**	
SM(d18:2/14:0)	Age	0.13	0.4	**<.001**
	BMI	-0.36	0.1	
	Time	241	**<.001**	
Cer(d18:1/24:0)	Age	-1.4	0.65	**<.001**
	BMI	-6.9	0.11	
	Time	-79	**0.003**	
Cer(d18:1/24:1)	Age	-2.5	0.07	**0.004**
	BMI	-1.4	0.39	
	Time	44.6	**0.02**	
SM(d18:1/22:1)	Age	0.56	0.7	**0.021**
	BMI	1.96	0.3	
	Time	671	**<.001**	
SM(d18:1/24:0)	Age	20.1	0.15	**<.001**
	BMI	-15.7	0.16	

	Time	-18	**0.001**	
GlcCer(d18:1/24:1)	Age	-0.17	0.48	**0.001**
	BMI	-0.39	0.19	
	Time	1342	**<.001**	
SM(d18:1/22:0)	Age	19.8	0.47	**<.001**
	BMI	-37.1	0.07	
	Time	-63.7	**0.04**	
LacCer(d18:1/16:0)	Age	0.55	0.77	**0.040**
	BMI	-1.18	0.65	
	Time	6	**0.009**	
SM(d18:0/14:0)	Age	-0.15	0.43	**0.011**
	BMI	-0.33	0.2	
	Time	-1010	**<.001**	
SM(d18:1/18:0)	Age	1.95	0.94	**<.001**
	BMI	-4.76	0.83	
	Time	327	**<.001**	
SM(d18:1/20:0)	Age	2.48	0.79	**<.001**
	BMI	-10.59	0.29	
	Time	-15	**0.01**	
SM(d18:0/18:0)	Age	-0.16	0.67	**0.011**
	BMI	0.3	0.57	

The GEE model considered two time points—before and after the procedure—as the main predictor, adjusting for age and BMI. P-values were adjusted for multiple comparisons using the Benjamini-Hochberg false discovery rate (FDR) method, with a significance threshold set at 3%, as adopted by MetaboAnalyst 6.0. Adjusted p-values are reported as q-values. Statistically significant p- and q-values are shown in bold.
