# Supplementary material for: Changes in Plasma Sphingolipid Metabolites Following Roux‐En‐Y Gastric Bypass in Women With Obesity and Type 2 Diabetes: A Pilot Metabolomic Cohort Study
Source: Lipids. 2025 Nov 12;61(2):195–205. doi: 10.1002/lipd.70019 (PMC12975409; doi:10.1002/lipd.70019)
Supplement: Supplementary file 5 — Appendix E Correlation analysis of sphingolipids with glycemic markers. [file LIPD-61-195-s005.pdf]

Appendix E. Correlation analysis between plasma sphingolipids and glycemic markers before and after Roux-en-Y gastric bypass

	Glucose mg/dL	Insulin µU/mL	HbA1c %	C peptide ng/mL
	**Pre-surgery (n=28)**			
**Cer(d18:1/23:0)**	0.36	-0.08	0.19	-0.23
**Cer(d18:1/24:0)**	0.20	-0.02	0.10	-0.20
**Cer(d18:1/24:1)**	0.19	-0.20	0.29	-0.52 **
**GlcCer(d18:1/24:1)**	0.27	0.04	0.25	-0.15
**SM(d18:1/12:0)**	-0.20	0.17	-0.53 **	0.27
**SM(d18:0/14:0)**	0.06	0.13	-0.13	0.01
**SM(d18:1/14:0)**	0.05	0.23	-0.16	0.19
**SM(d18:2/14:0)**	-0.18	0.28	-0.56 **	0.26
**SM(d18:1/18:0)**	-0.07	0.05	0.10	-0.30
**SM(d18:1/20:0)**	0.02	0.11	-0.06	-0.07
**SM(d18:1/21:0)**	0.15	0.08	-0.05	-0.01
**SM(d18:1/22:0)**	0.04	0.03	0.06	-0.15
**SM(d18:1/22:1)**	-0.11	-0.15	-0.13	-0.11
**SM(d18:1/23:0)**	0.18	0.04	0.11	-0.03
**SM(d18:1/24:0)**	0.04	0.15	0.09	-0.04
**SM(d18:2/24:1)**	-0.26	0.00	-0.04	-0.13
	**Post-surgery (n=28)**			
**Cer(d18:1/23:0)**	0.30	-0.11	0.46 *	0.10
**Cer(d18:1/24:0)**	0.27	-0.18	0.44 *	-0.04
**Cer(d18:1/24:1)**	0.14	-0.10	0.37	0.15
**GlcCer(d18:1/24:1)**	-0.03	-0.06	0.38	0.08
**SM(d18:1/12:0)**	0.31	0.15	0.22	-0.09
**SM(d18:0/14:0)**	0.18	0.22	-0.07	-0.11
**SM(d18:1/14:0)**	0.29	0.08	0.49 *	-0.02
**SM(d18:2/14:0)**	0.20	0.09	0.28	-0.19
**SM(d18:0/18:0)**	-0.20	0.04	-0.01	-0.21
**SM(d18:1/18:0)**	-0.16	-0.28	0.48 *	-0.08
**SM(d18:1/20:0)**	0.24	-0.19	0.49 *	-0.19
**SM(d18:1/21:0)**	0.25	-0.09	0.48 *	-0.06
**SM(d18:1/22:0)**	0.15	-0.36	0.49 *	-0.20
**SM(d18:1/22:1)**	0.03	-0.19	0.21	-0.31
**SM(d18:1/23:0)**	0.20	-0.33	0.46 *	-0.20
**SM(d18:2/23:0)**	0.09	-0.07	0.43 *	-0.04
**SM(d18:1/24:0)**	0.09	-0.42 *	0.39	-0.28
**SM(d18:2/24:0)**	-0.14	-0.58 **	0.22	-0.38
**SM(d18:2/24:1)**	-0.28	-0.42 *	-0.18	-0.45 *

This table shows Spearman's correlation coefficient of sphingolipids analyzed and plasma glycemic markers. Negative correlations are presented with a minus sign (-) in red color, and positive correlations are expressed in blue color. The more intense colors in the boxes represent stronger correlations. *p < 0.05; **p < 0.01; p < 0.001.
